# Glioblastoma multiforme in conus medullaris with intracranial metastasis after postoperative adjuvant therapy

**DOI:** 10.1097/MD.0000000000006500

**Published:** 2017-03-31

**Authors:** Chengrui Yan, Xiangyi Kong, Hua Yin, Yu Wang, Huayu He, Hui Zhang, Jun Gao, Yongning Li, Wenbin Ma

**Affiliations:** a Department of Neurosurgery, Peking Union Medical College Hospital, Chinese Academy of Medical Sciences, No. 1 Shuaifuyuan, Dongcheng District, Beijing, P.R. China; b Department of Anesthesia, Critical Care and Pain Medicine, Massachusetts General Hospital, Harvard Medical School, Harvard University, MA; c Department of Pathology, Peking Union Medical College Hospital, Chinese Academy of Medical Sciences, No. 1 Shuaifuyuan, Dongcheng District, Beijing, P.R. China.

**Keywords:** conus medullaris, immunohistochemistry, predictive factor, spinal glioblastoma multiforme, treatment

## Abstract

Spinal glioblastoma multiforme is not common among spinal cord tumors. According to our literature review, only 27 cases originating from the conus medullaris were reported. We herein reported a case of a 10-year-old child diagnosed with glioblastoma multiforme. The patient received adjuvant radiotherapy and standard temozolomide chemotherapy after total excision. Intracranial lesions were found 1 month after postoperative adjuvant therapy. We described the clinical characteristics and postoperative therapy of the patient, and reviewed all of the published cases of conus medullaris glioblastoma. Location, age, leptomeningeal spread, and secondary hydrocephalus may be predictive factors. Immunohistochemical factors such as p53 and Ki-67 are also important. Combined treatment of surgery and postoperative adjuvant therapy is commonly used, but is controversial.

## 1 Introduction

In adults, spinal cord tumors mostly originate from extramedullary tumors (almost 80%),^[1]^ whereas in children, the rate of primary spinal tumor is up to 35%.^[2]^ Among these neoplastic spinal lesions in children, high-grade glioma is relatively rare (roughly 1%-3%). Spinal glioblastoma multiforme (GBM), defined as World Health Organization (WHO) IV in astrocytoma, is a highly malignant central nervous system (CNS) tumor that is clinically, histologically, and genetically heterogeneous.^[3]^ A survey in 1989 revealed that spinal GBM accounted for 0.2% of all GBM and 1.4% of spinal glioma.^[4]^ The total number of cases reported in the literature was less than 200.^[5]^ According to the data in a single institution, spinal cord GBM accounted for only 1% of patients with intramedullary neoplasms.^[5]^ Spinal GBM accounted for 7.5% of intramedullary glioma, and only 1.5% of all spinal tumors.^[6]^ As we present a rare case of GBM located in conus, an effort was made to search for cases of conus GBM published ever (Table 1), to detect some similarities and to learn more about metastasis, pathology, and treatment.

**Table 1:**
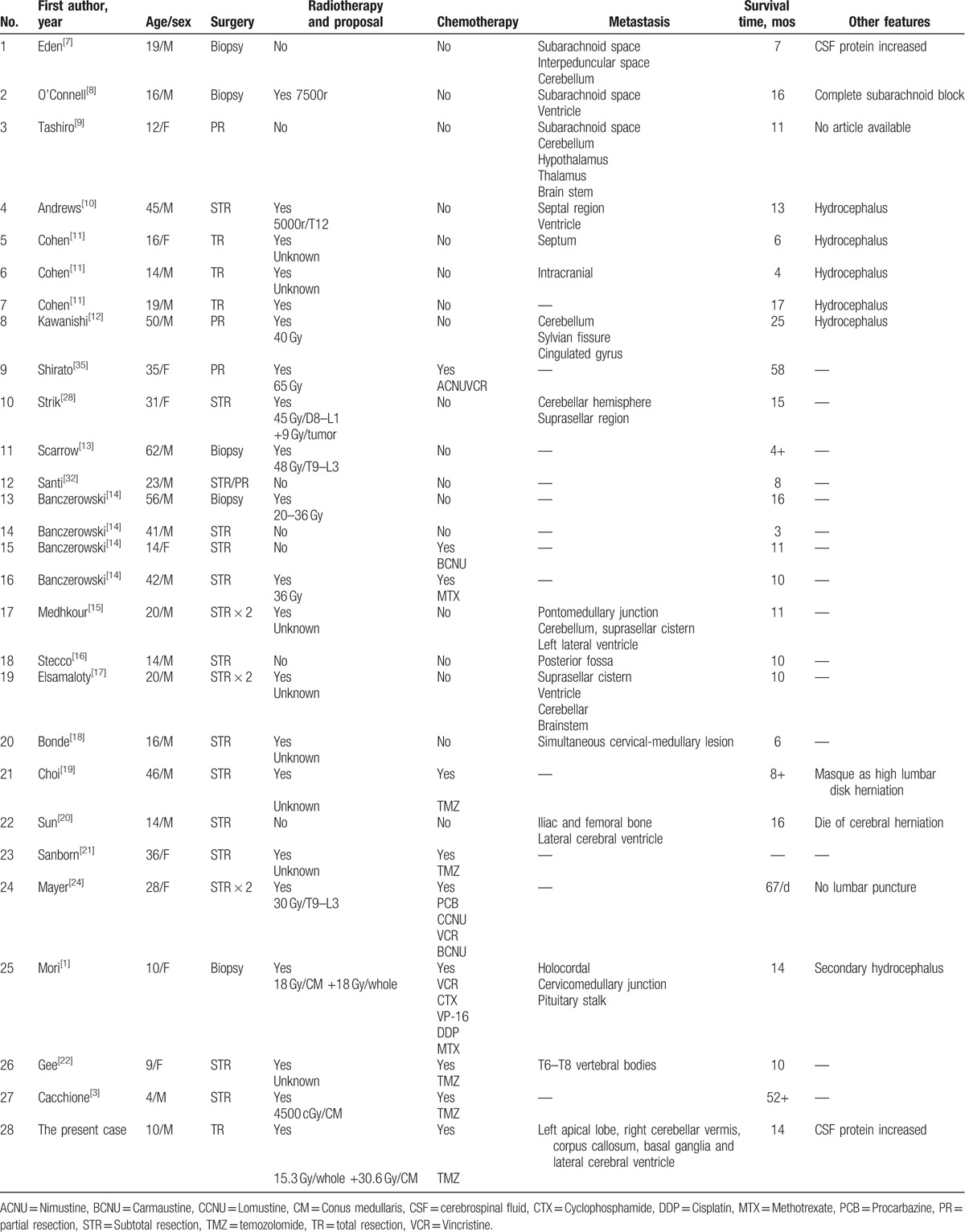
Clinical features of conus medullaris glioblastoma reported in previous literatures.

## 2 Case presentation

A 10-year-old Chinese boy, without any past medical history, reported a 3-week history of weakness and a subsequent appearance of paresthesia of both lower limbs. He also complained of lower back pain with radiation to both legs, urinary disturbance, and weight loss. His examination of strength demonstrated a 3/5 at right leg and 4/5 at left leg. Sensory examination was significant for decreased sensation in both legs, whereas right leg was worse. His reflex was weakened at left patella and ankle, and was absent at right. Bilateral hyperexplexia were presented of the lower limbs. There were no neurologic abnormalities of the upper limbs and cranial nerves.

**Figure 1:**
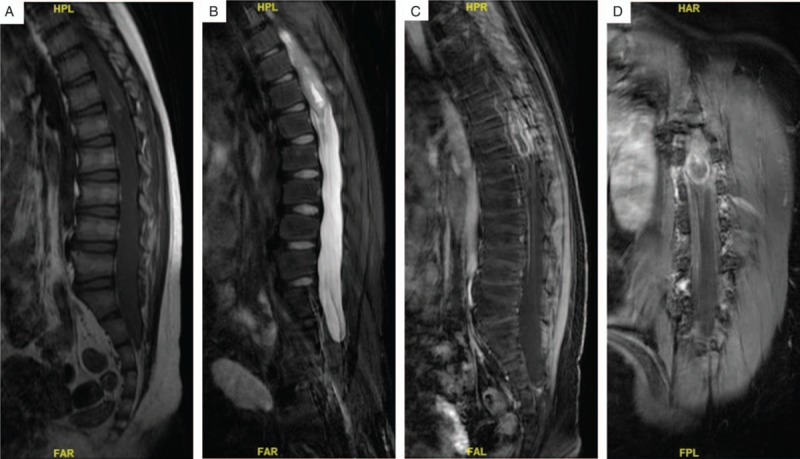
(A, B) T1-weighted and T2-weighted sagittal magnetic resonance imaging (MRI) without contrast shows an intramedullary lesion extended from T11 to L1. (C) T1-weighted sagittal MR image with contrast reveals heterogeneous enhancement of the conus lesion. (D) T1-weighted coronal MR image with contrast.

Magnetic resonance imaging (MRI) revealed an ill-defined intramedullary mass filled the spinal canal between T11 and L1 (Fig. 1A and B) with inhomogeneous enhancement of the tumor area (Fig. 1C and D). Based on these findings, glioma was considered.

Open total excision was conducted under general anesthesia. The procedure presented a hypertonic dura. After opening the dura, the tumor was located in the conus medullaris, highly vascularized, and ill-defined.

**Figure 2:**
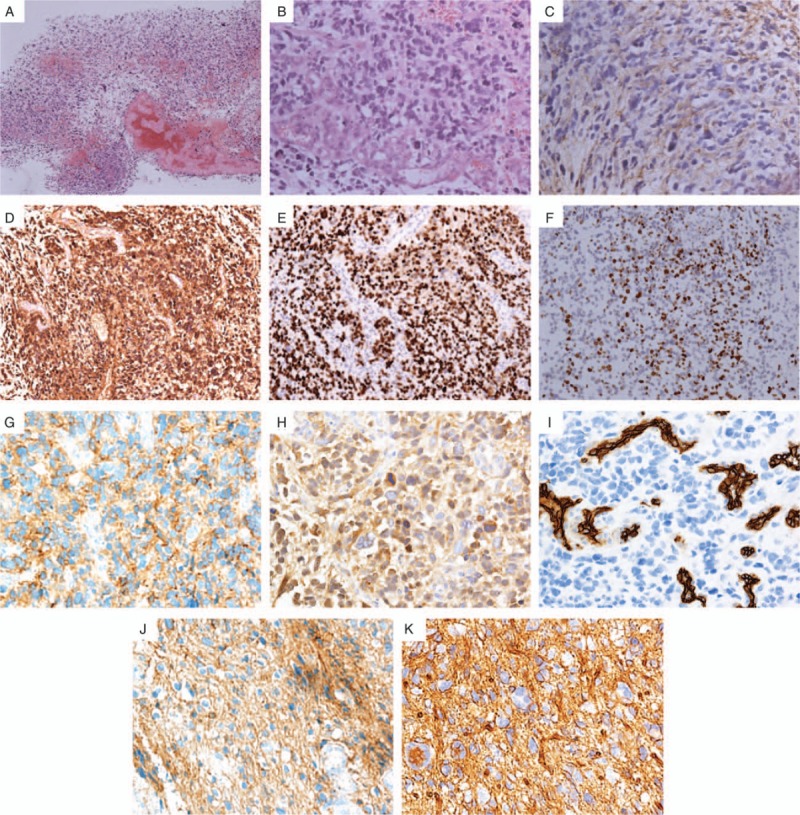
(A) Tumor cells were pleomorphic and patchily distributed (H&E stain; 20×). (B) Densely cellular neoplasm, with multiple pleomorphic cells, proliferation of vascular endothelial cells, and karyokinesis (H&E stain; 200×). (C) Immunohistochemistry shows positivity for glial fibrillary acidic protein (GFAP). (D) The tumor shows diffused S100 positivity. (E) The tumor shows positivity of p53. (F) The tumor shows a high Ki-67 immunohistochemical nuclear labeling index of 60%. Epidermal growth factor receptor (EGFR) (G), NSE (H), CD34 (I), CD56 (J), and β-catenin (K) were also positive (20×).

Histopathologic examination, including immunohistochemical staining, was performed, and the diagnosis of GBM was made according to the WHO criteria (Fig. 2A and B). However, part of the tumor showed the structure of primitive neuroectodermal tumor (PNET). Immunohistochemistry showed positivity for glial fibrillary acidic protein (GFAP) (Fig. 2C), S100 (Fig. 2D), p53 (Fig. 2E), Ki-67 (proliferation index 60%; Fig. 2F), epidermal growth factor receptor (EGFR) (Fig. 2G), neurone-specific enolase (NSE) (Fig. 2H), CD34 (Fig. 2I), CD56 (Fig. 2J), and β-catenin (Fig. 2K), and negativity for chromograin A (CgA), AE1/AE3, CD99, and neurofilament (NF). The mutation or amplification of EGFR is commonly observed in malignant gliomas, and these modifications are associated with increased cell proliferation and radiation resistance. NSE is a specific protein of neuron; high serum levels of NSE were noticed in the patients with malignant gliomas. CD34 and CD56 reflect densities of vessel and blood supply, whose overexpressions are associated with higher WHO grades of gliomas. CD34 may serve as a potential diagnostic and prognostic marker, or it could be a useful therapy target. Beta-catenin is a proto-oncogene. Mutations of this gene are commonly found in a variety of cancers, including GBM. CD99 is more expressed in malignant gliomas than in the brain, and such overexpression results in higher levels of invasiveness and lower rates of survival.

The plain and enhanced MRI of head revealed no abnormality. Lumbar puncture was also conducted, and the protein concentration in the cerebrospinal fluid (CSF) was high and the glucose concentration was low. No malignant cells were found in the CSF.

The patient underwent radiotherapy for the whole brain and spinal cord (15.3 Gy in 9 fractions), and also for the conus medullaris lesion (T8–L4, 30.6 Gy in 18 fractions). As for chemotherapy, standard temozolomide (TMZ)-stupp regimen (TMZ 150 mg/m^2^ for 5 days during first 28-day and 200 mg/m^2^ for 5 days during next sixth 28-day cycles) was performed. After these adjuvant therapies, this patient showed improved length of 4/5 at right leg and 5/5 at left leg. Meanwhile, reflex of left patella and ankle recovered, and reflex of right leg became better.

**Figure 3:**
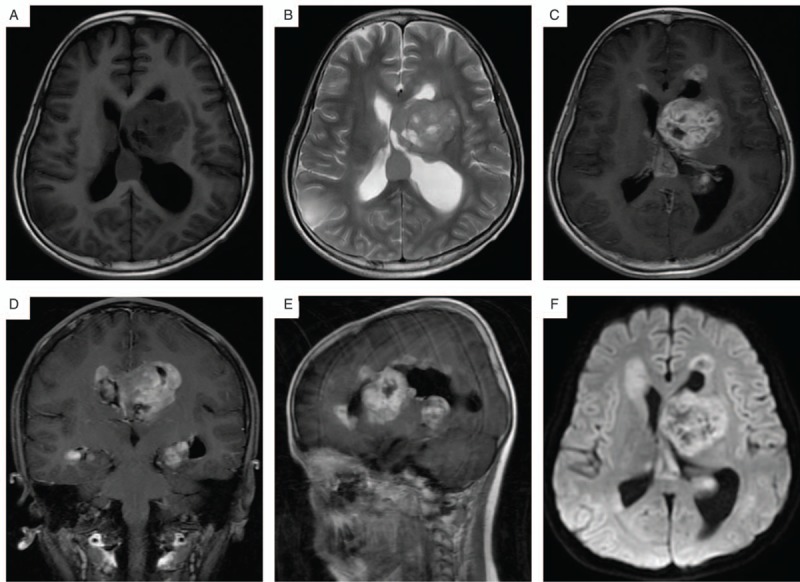
Follow-up magnetic resonance imaging (MRI) after 10 months from operation demonstrates intracranial metastasis. (A, B) T1-weighted and T2-weighted axial MR images show new lesions located in lateral cerebral ventricle and enlargement of ventricle. (C) T1-weighted sagittal MR image with contrast reveals heterogeneous enhancement. (D, E) Coronal and sagittal sections display several metastases. (F) Apparent diffusion coefficient (ADC) mapping shows heterogeneous signal.

After 1 month from last regimen of chemotherapy (10 months from operation), a follow-up MRI of head with gadolinium enhancement revealed multiple intracranial metastasis. New lesions were located in left apical lobe, right cerebellar vermis, corpus callosum, basal ganglia, and lateral cerebral ventricle (Fig. 3). We deemed them as the metastatic lesions from the tumor in conus medullaris. The patient and his parents refused further invasive treatments; thus the exact pathology of the intracranial lesions could not be confirmed. The patient died 14 months after his surgery.

## 3 Discussion

Primary spinal GBM located in conus medullaris is relatively rare; usually this kind of disease has a favor for the thoracic, cervical, or the conjunction area. In a literature review in 2011, the author found that conus medullaris is also a location where spinal GBM may generate, although it is not that usual.^[21]^ A study retrospect 128 cases from 1938 to 2015 performed that 42.2% of spinal GBM were located in thoracic spine and 29.7% were in cervical spine. Meanwhile, tumors located in the conus level only accounted for 14%, according to this study.^[23]^

Generally, the survival time of spinal GBM was limited to 12 to 24 months. Moreover, GBM of the conus medullaris ranged from 4 to 16 months. The mean follow-up time (most are equal to survival time) in our study was 18.26 months, among which the longest one was 67 months.^[24]^ Konar et al^[23]^ found that location of the tumor had no influence to overall survival, and patients with conus and thoracic tumors were less likely to die at 6 months. As to the influence of age, people have same opinions that adults (over 18 years) were more likely to have longer survival periods.^[23,25]^ But Wolff et al^[26]^ found that in children, age less than 5 years may be a relatively positive prognostic factor.

Among these predictive factors, Konar et al thought leptomeningeal spread is the poor prognostic factor for survival. The rate of CSF dissemination reaches up to 58% in spinal GBM, in comparison with 23% to 27% in cerebral GBM.^[27]^ In Konar et al's study, 60% of the patients had CSF dissemination and 76% had brain metastasis.^[23]^ We found that in those 28 cases of conus GBM, 16 cases mentioned tumor metastasis, and among these, 14 patients had brain metastasis. It is easily recognized that brain metastasis occurred in most spinal GBM. Patchy nodular lesions were discovered intracranially, whereas the specific locations differed. According to our unpublished study, wall of the ventricles could become a relatively welcome “home” of new lesions, considering the anatomy. Strik et al^[28]^ noted that p53 may be a predictor of subsequent brain metastasis in spinal GBM. But we lack more evidence about p53 accumulation for the reason that few cases conducted immunohistochemical study. Of note, regarding the pathology of the primary lesion in our case, part of it showed the structure of PNET. In fact, primary PNET of the spine is unusual, with very few cases reported in the literature. Occurrence of primary spinal PNET in an intramedullary location is further uncommon. Because the patient and his parents refused further invasive treatments after the discovery of intracranial lesions, their exact pathology could not be confirmed.

Another important factor of overall survival period is secondary hydrocephalus. Six of the 28 patients in our study developed hydrocephalus. Higher concentration of protein in the CSF was thought to be related with hydrocephalus. Our patient also demonstrated to contain more protein in CSF, but he did not develop hydrocephalus. It is possible that the tumor had not blocked the subarachnoid completely. Most of the cases we found did not perform a lumbar puncture or did not display the results, but we can speculate that higher protein may be a sign of CSF dissemination.

For the reason that spinal GBM was not common in population, studies describing the histomorphological and molecular genetic alterations are not as many as cerebral GBM. Govindan et al^[29]^ had the opinion that histopathological characteristics of spinal GBM are comparable with cerebral glioblastoma. In their 6-case study, GFAP was 100% positive, whereas p53 immunoreactivity was 83.3% (5 of 6).^[29]^ Another case series study concluded that GFAP and p53 immunoreactivity was seen in all cases.^[30]^ Studies also showed that p53 expression is seen in majority of glioblastoma. The pathology of our case was definitely positive for GFAP and p53, matching with results of those research. Eleven patients had immunochemical results in the list, and 8 of them expressed GAFP, but only 4 mentioned reactivity of p53. Even though the expression of p53 was not as expected, we are of the opinion that it may be a result of lack of data. Proliferative marker Ki-67 index/MIB-1 labeling should be a complement of pathologic features of GBM. It has been reported that Ki-67 index ranged from 12% to 34%.^[30]^ The Ki-67 index ranged from 10% to 30% in our case series, with the values being comparable with those of other studies.^[31]^ However, the boy in our case showed a very high Ki-67 index of 60%. It may be a hint why the patient had brain metastasis after radiochemotherapy, even though the prognostic value of Ki-67 was debatable.^[32]^

Treatment of spinal GBM nowadays usually combines surgery with radiotherapy; most of the time chemotherapy is also considered. As for radiotherapy, although researchers did not find significant linkage between radiation and prognosis,^[33]^ more people believe radiation can increase survival time in malignant spinal tumor.^[25]^ But the optimal dosage is uncertain, although it is almost always used. It is reported that radiotherapy can prolong survival in some cases.^[34]^ Shirato et al^[35]^ recommended that radiation can be given in 2.5-Gy fractions 4 times weekly to total doses of 40 to 50 Gy over 4 to 5 weeks. Our patient received a total dose of 45.9 Gy for conus medullaris and an additional radiation to the whole brain. However, the tumor still metastasized to intracranial spaces. If patient tolerates the treatment well, Minehan et al^[36]^ found that higher doses (59.4 Gy) could perform better in symptom improvement. Shirato et al also reported a long survival case for 58 months with a total radiation dose of 65 Gy, but in consideration of adverse effects on growing and fertility, especially in teenage, we have to control the total dose of radiation.

Whether chemotherapy is required or not remains controversial, but a retrospective series of 8 cases proved that both TMZ and bevacizumab were useful in improving survival.^[37]^ As TMZ was recognized to be effective in intracranial GBM,^[38]^ it is also generally used as adjuvant therapy to surgery and radiation in spinal GBM. For patients with spinal GBM, it is recommended that TMZ be used concomitantly during and after radiotherapy, but at different dosage. In a study consisting of 6 patients, TMZ seemed to prolong survival time of primary spinal GBM.^[39]^ A patient without CSF dissemination received 7 cycles of TMZ,^[41]^ the same regimen as in the patient described in our case. Unfortunately, both these patients revealed new intracranial lesions during follow-up. Our patient did not receive any salvage therapy, but the outcome was exactly the same as that of the patient mentioned. Despite the discouraging results, Konar et al's analysis revealed that surgical combined radiotherapy and chemotherapy were significantly associated with a significant chance of mortality at 6 months.^[23]^ Chamberlain and Johnston^[40]^ found bevacizumab may have some effects in those who failed to response to radiation and TMZ therapy. In our analysis, patients who received either adjuvant radiotherapy or chemotherapy had a better survival trend than those with surgery alone.

With the development of molecular therapy, tumors can be investigated more and more thoroughly. In a study by Sharma et al,^[41]^ it was shown that children may have different molecular signature from adults. So we can have the confidence that target therapies may become a powerful measure in treating spinal GBM.

## 4 Conclusions

Spinal GBM located in conus medullaris is rare. Several factors may be related to intracranial metastasis and prognosis. Immunohistochemistry currently plays a crucial role in diagnosis of CNS tumors. Adjuvant treatment composed of radiotherapy and chemotherapy are still under exploration.
